# Proteasome inhibition rapidly exacerbates photoinhibition and impedes recovery during high light stress in *Chlamydomonas reinhardtii*

**DOI:** 10.1186/s12870-020-2236-6

**Published:** 2020-01-13

**Authors:** Felipe Mendoza, Carson Berry, Laura Prestigiacomo, Doug Van Hoewyk

**Affiliations:** Coastal Carolina University, 113 Chanticleer Dr, Conway, SC 29528 USA

## Abstract

**Background:**

Proteasomes remove regulatory proteins in eukaryotic cells, and control a variety of plant processes. Proteasomes are localized to the cytosol and nuclear, but their role in plant biology has recently been extended to chloroplasts, where it regulates TOC complex. This is turn controls the import of nuclear-encoded chloroplastic proteins, which remodels the chloroplast proteome and facilitates proper developmental transitions. Proteasomal regulation of the TOC complex also alleviates stressors that generate reactive oxygen species. These recent advances motivated us to determine if proteasome inhibition rapidly alters photosynthetic processes stemming from photoinhibition induced by high light.

**Results:**

The short-term effects of proteasome inhibition on photosystem II during light stress was measured in *Chlamydomonas reinhardtii*, which allowed the dual monitoring of both chlorophyll fluorescence and cell viability. After 48 h at low light, proteasome inhibition did not affect viability or photochemistiry, but decreased cell concentration and increased cell volume. Two hours of high light stress impaired the efficiency of photosystem II in proteasome-inhibited cells, as determined by a decrease in Fv/Fm and the electron transport rate. Elevated photoinhibition in proteasome inhibited cells was not caused by a decrease in cell viability or chlorophyll content. Recovery from photoinhibition was attenuated in MG132-treated cells, and suppressed growth of a reestablished culture. Proteasome inhibition decreased de novo protein synthesis, which possibly constrained the ability to remodel the plastid proteome, and thus hampering the ability to adjust to high light stress.

**Conclusion:**

The proteasome is implicated in protecting photosystem II from photoinhibition. In addition to high light stress, other stressors- including metals, drought, and salt- are also known to generate reactive oxygen species localized to the chloroplast. Therefore, proteasome maintenance in plants may help protect photosynthesis during abiotic stress, which could increase crop yield during adverse conditions.

## Background

Optimal development and growth in plants depends on their ability to adjust photosynthesis during environmental constraints. Threats encountered by plants- including salt, heavy metals, or drought- generate reactive oxygen species (ROS) in various cellular compartments, including chloroplasts [1]. High light (HL) stress can also produce ROS that overwhelm the antioxidant capacity in chloroplasts, which can lead to decreased photochemistry in a process called photoinhibition [2]. Reduced photochemistry constrains crop yield, and can dictate the ability of plants to survive in challenging environments.

Both photosystem (PS) II and PSI generate ROS during HL stress. Superoxide can damage the iron-sulfur clusters in proteins found predominantly in PSI [3] and ferredoxin [4]. Compared to PSI, however, PSII is more susceptible to photoinhibition, and includes the production of singlet oxygen and superoxide [5]. To counter the deleterious effects of photoinhibition, chloroplasts can make various adjustments during HL stress; well-documented changes in PSII include decreasing antenna size, repairing damaged D1 proteins in the PSII reaction core, non-photochemical quenching, and thylakoid unstacking [6]. In additional to changes in PSII, an efficient stress response in chloroplasts necessitates proteome remodeling. Because ~ 90% of chloroplastic proteins are nuclear encoded, plastid proteome adjustments during stress require the proper import of precursor proteins from the cytosol via the TIC/TOC (translocon at the outer/inner envelope of chloroplasts) protein complex [7].

On a cellular level, stress-induced proteome adjustments are governed by both transcriptional reprogramming and protein degradation [8]. Selective protein degradation is controlled by the ubiquitin-proteasome pathway (UPS) [9]. During this process, target proteins are tagged with the protein ubiquitin which requires the coordinated action of E1 ubiquitin activating enzymes, E2 conjugating enzymes, and E3 ubiquitin ligases; collectively, this post-translational modification delivers ubiquitinated proteins to proteolytic proteasomes located in the nucleus or cytosol. Proteasomes remove short lived regulatory proteins that govern various cellular processes, including cell division [10], nutrient uptake [11], microtubule assembly [12] (Wang et al., 2011), and hormonal signaling that can regulate photomorphogenesis and a stress response [13, 14]. Additionally, proteasomes remove damaged (e.g. oxidized or misfolded) proteins that result from stress, including misincorporation of selenocysteine in proteins [15]. Severe stress, however, can decrease proteasome activity in plants, such as salt [12, 16] heavy metals, and high light intensity [17]. Proteasome impairment likely stems from stress-induced ROS accumulation as observed in well-studied mammalian systems [18] and Chlamydomonas [19].

Even though chloroplasts do not contain proteasomes, the UPS is nonetheless implicated in plastidic processes. Recently, the E3 ligase SP1 was discovered to localize to the outer membrane of chloroplasts [20], where it controls the ubiquitination of TOC protein machinery. Arabidopsis plants with mutations in SP1 have altered thylakoid stacking and chloroplast biogenesis during early stages of development and senescence, which was explained by the inability of the TOC complex to properly control the import of proteins required during developmental transitions. Mutation in SP1 also decreased chlorophyll content upon being transferred from dark to continuous light. Additionally, SP1 also has a role in maintaining chloroplast proteome poise during abiotic stress [21]. Arabidopsis plants overexpressing SP1 have enhanced tolerance to stressors that generate ROS, including paraquat which induced photoinhibition by generating superoxide in chloroplasts. Stress was exacerbated in SP1 mutants, which was explained by their inability to reduce the TOC complex; the subsequent inability to regulate protein import during stress led to ROS accumulation in plastids and increased sensitivity to salt and paraquat. However, high light stress failed to produce a phenotype in plants with altered levels of SP1.

Proteasomes are also mediate the removal of damaged chloroplasts that result from stress-induced ROS accumulation. In this process, the cytosolic E3 ligase PUB4 tags impaired chloroplasts [22] that are removed in a process termed chlorophagy. Compared to wildtype Arabidopsis plants, the *pub4* mutants have more chloroplasts under control conditions, and exhibit significant growth retardation under high light.

Collectively, these recent advances have begun to unravel a role for proteasomes in optimizing chloroplast processes during stress or developmental transitions. Arabidopsis plants with mutations in proteasome assembly have developmental delays when exposed to continuous light [23], and mechanisms have now been proposed that can account for these observations. Given the pleotropic effects caused by proteasome inhibition, delineating how proteasomes impact phytochemistry remains a challenge. For example, it is not known if proteasomes protect against the deleterious effects of photoinhibition during light stress, which generates singlet oxygen. However, potentially implicating the involvement of proteasomes during light stress, *Chlamydomonas* treated with the photosensitizer neutral red produced singlet oxygen and increased 14 transcripts encoding proteasome subunits within two hours [24].

The goal of this study was focused on determining if photosynthetic efficiency in PSII is altered in proteasome-inhibited *Chlamydomonas reinhartdtii* cells challenged by high light stress. We sought to determine if exacerbated photoinhibition in proteasome-inhibited cells occurred prior to decreased viability or chlorophyll content. Another objective of this study was to determine if PSII recovery from photoinhibition was delayed in proteasome inhibited cells, and if this would alter subsequent growth of the population. This study reveals a role for proteasomes in achieving optimal photosynthetic efficiency during photoinhibition, and we discuss how this data can be integrated into a broader understanding of plant stress physiology.

## Results

**Fig. 1:**
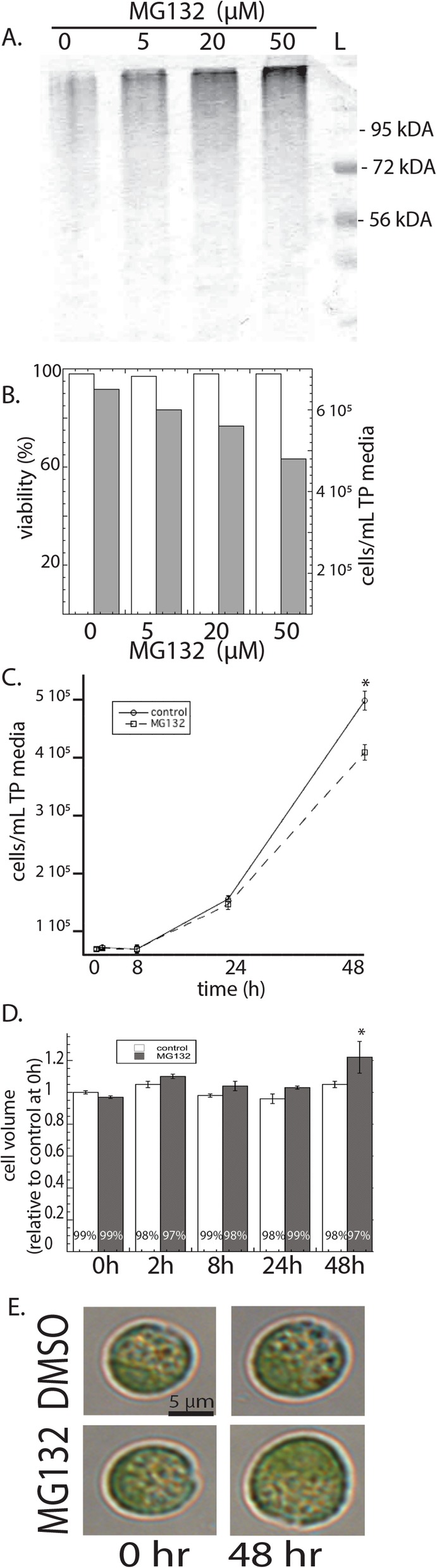
The effects of the proteasome inhibitor MG132 in Chlamydomonas. **a** The effect of 0, 5, 20, and 50 μM MG132 on levels of ubiquitinated proteins after 48 h of treatment were evaluated on SDS-PAGE electrophoresis. **b** Chlamydomonos were treated with 20 μM MG132 for 48 h, at which point viability and cell concentration were determined via flow cytometety. White and black columns represent viability and cell concentration, respectively, on the left and right axes. **c** The effect of 20 μM MG132 on cell concentration in Chlamydominas cultures were determined at different time points (0, 8, 24, and 48 h). **d** Cell volume was determined in cells treated with or without 20 μM MG132 at different time points. **e** The effect of 20 μM MG132 on cell volume; cells were grown for 48 h with or without MG132 and subsequently imaged using light microscopy. Shown are the means and standard errors of five replicate cultures, which are representative of two other experimental replicates. Asterisks represent a significant difference (*p* < 0.05) between treatments at each time point. L-ladder, kDA- kilodalton

We initially wanted to determine the effects of the proteasome inhibitor MG132 on the growth of Chlamydomonas in order to establish that it is toxic. Cultures (10^5^ cells ml ^− 1^) were treated with 0, 5, 20, and 100 μM MG132 for 2 days. Ubiquitinated proteins accumulated in MG132-treated cells in a dose-dependent manner, demonstrating the efficacy of the proteasome inhibitor (Fig. 1a). Proteasome inhibition did not affect viability, but decreased rates of cell division as determined by cell concentration (Fig. 1b). All subsequence experiments used 20 μM MG132, because this concentration sufficiently inhibited the proteasome without drastically decreasing cell division after 48 h. Further analysis revealed that a 20 μM MG132 did not alter population growth after 8 or 24 h (Fig. 1c). At 48 h, MG132 decreased cell concentration, but increased the average cell volume by 20% compared to untreated cells (Fig. 1d,e).

**Fig. 2:**
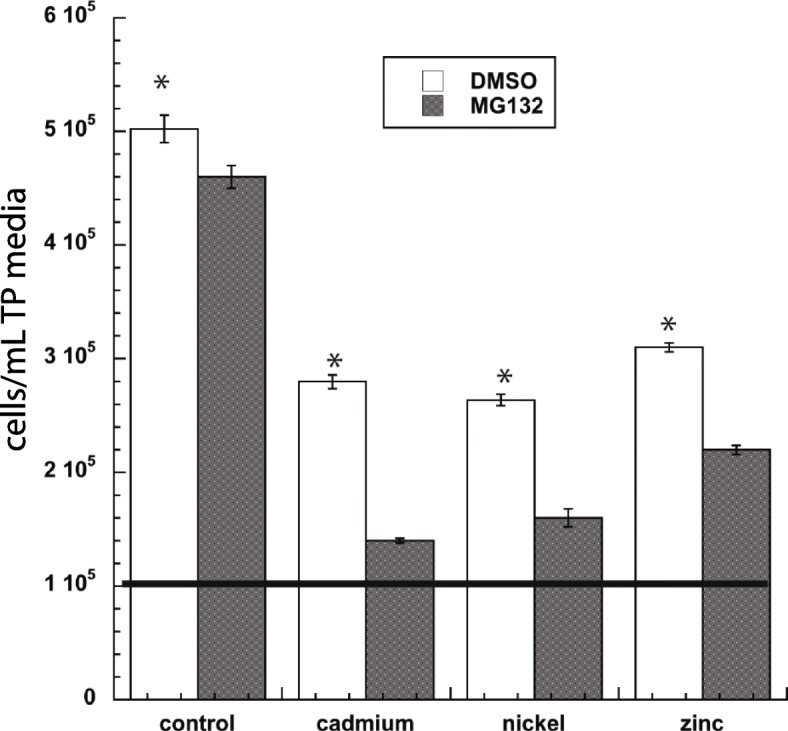
The effect of 20 μM MG132 on cell concentration was evaluated in cells challenged with metals. Cultures initially contained 10^5^ cells (represented by the horizontal line) and were either untreated or treated with cadmium, nickle, or zinc; cell concentration was measured 48 h later. Shown are the means and standard errors of five replicates, which are representative of at least two other experimental replicates. Asterisks represent a significant difference (*p* < 0.05) between treatments at each time point

To demonstrate the consequences of proteasome inhibition in *Chlamydomonas* challenged with stressors that induces oxidative stress, cells were grown with or without MG132 for 2 h and then treated with nickel, cadmium, or zinc for two days. Proteasome inhibition greatly increased sensitivity to the metals, as determined by a decrease in cell concentration (Fig. 2). However, these metals are known to induce ROS localized to the cytosol, chloroplast, and mitochondria, and therefore impede various metabolic processes in addition to photosynthesis. Therefore, an analysis on PS II efficiency was not performed.

**Fig. 3:**
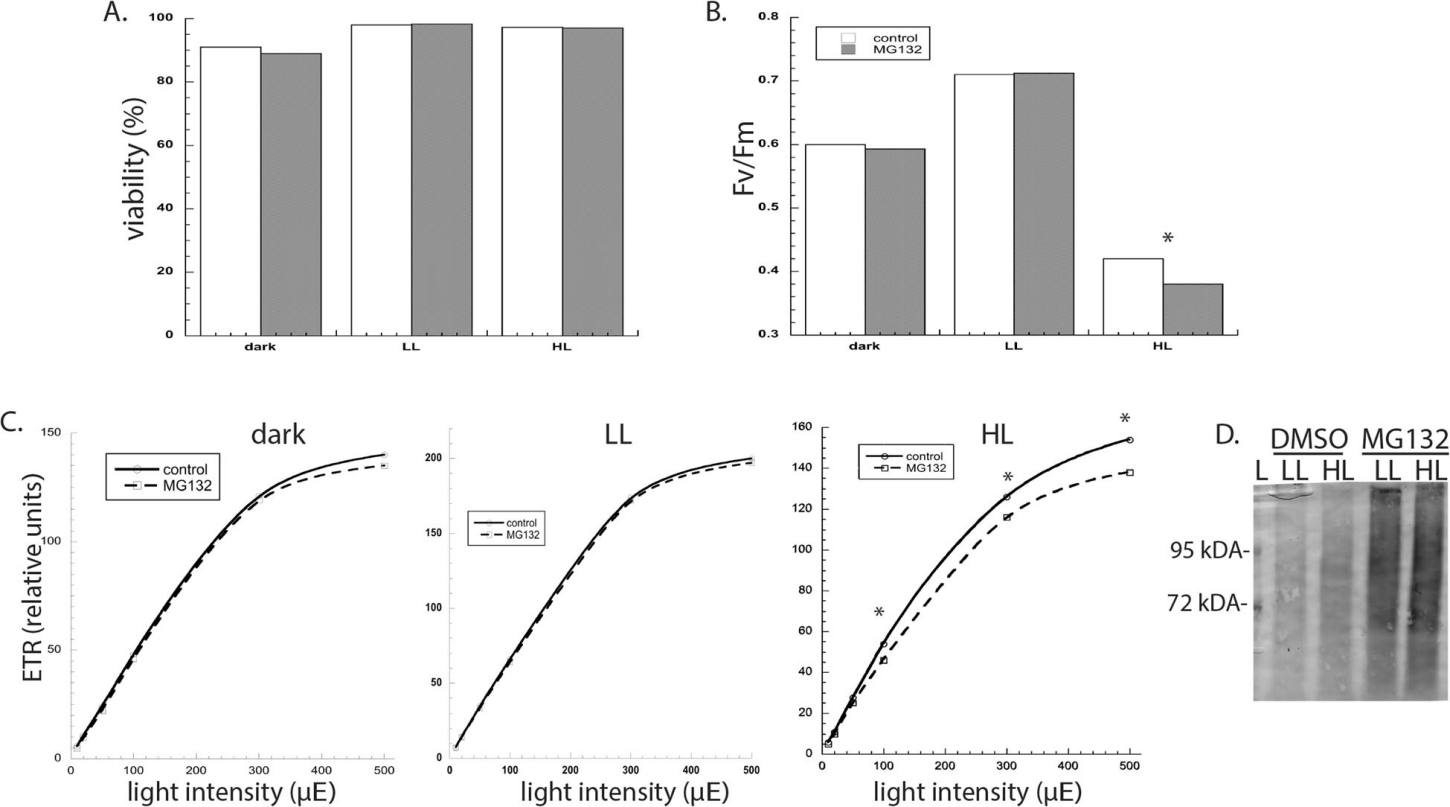
The effects of proteasome inhibition on the (**a**) viability, **b** maximum photochemical efficiency of PSII (Fv/Fm), and **c** ETR of Chlamydomonas cells grown at three different light conditions. Measurements were taken 48 h for algae grown in the dark or low light, or 2 h for algae grown under high light. **d** The effect of 20 μM MG132 was evaluated in cells grown under low and high light for 4 h. Shown are the means and standard errors of five replicate cultures, which are representative of at least five other experimental replicates for chlorophyll fluorescence measurements and two experimental replicates for flow cytometry measurements. Error bars are too small to plot. Asterisks represent a significant difference (*p* < 0.05) between treatments at each time point. L-ladder; LL- low light; HL- high light; ETR- electron transport rate

Rather, we focused our experiments on understanding the short-term effects of proteasome inhibition on photosynthesis in cells that with different light regimes. Cultures were grown +/− MG132 in LL, and then transferred in the dark and low light (LL) for 24 h, or high light (HL) for 2 h. Although dark treatment decreased cell concentration compared to LL conditions, MG132 treatment did not antagonize cell survival in any of the conditions, as viability ranged from 97 to 99% (Fig. 3a). After 24 h of darkness or LL, proteasome inhibition did not affect Fv/Fm values, which measures the efficiency of photochemical reactions in photosystem II; additionally, there was no difference in the electron transport rate (ETR) at varying light intensities (Fig. 3b,c). In contrast, MG132 treatment exacerbated the effects of photoinhibition. Growth at HL decreased both Fv/Fm and ETR in cells after two hours. Western blot analysis confirmed that levels of ubiquitinated proteins accumulated during this time frame (4 h), thus confirming MG132 efficacy; levels of ubiquitinated proteins was not affected by HL (Fig. 3d).

Optimal efficiency of photosynthesis is strongly associated with chlorophyll content. An objective of this study was to determine if decreased Fv/Fm values in proteasome-inhibited cells at HL for two hours occur independently of changes in chlorophyll content. As expected, HL decreased chlorophyll a and b compared to cells grown in LL, but did not affect the chlorophyll a:b ratio (Table 1). However, four hours of proteasome inhibition did not alter chlorophyll content in cells grown at LL or HL. Additionally, viability was not altered in cultures subjected to different conditions. MG132-treated cells increased protein content; this result was anticipated because proteasome-mediated proteolysis was inhibited.

**Table 1:** Viability, protein content, and chlorophyll concentrations of cultures with or without MG132 grown at LL or HL for 2 h. Different letters represent a significant difference (*p* < 0.05) between treatments

	Viability (%)	protien (ug/mL)	chlorophyll a (ug/ml)	chlorophyll b (ug/ml)	chl a: chl b
LL	98.3(0.5)^a^	0.43(0.06)^a^	2.12 (0.13)^a^	0.87(0.1)^a^	2.43 (0.15)^a^
LL-MG132	98.7 (0.9)^a^	0.62 (0.07)^b^	2.07 (0.05)^a^	0.81 (0.07)^a^	2.55 (0.07)^a^
HL	97.6 (1)^a^	0.41 (0.03)^a^	1.77 (0.11)^b^	0.69 (0.15)^b^	2.56 (0.22)^a^
HL-MG132	98.1 (0.8)^a^	0.67 (0.01)^b^	1.8 (0.09)^b^	0.68 (0.06)^b^	2.64 (0.16)^a^

**Fig. 4:**
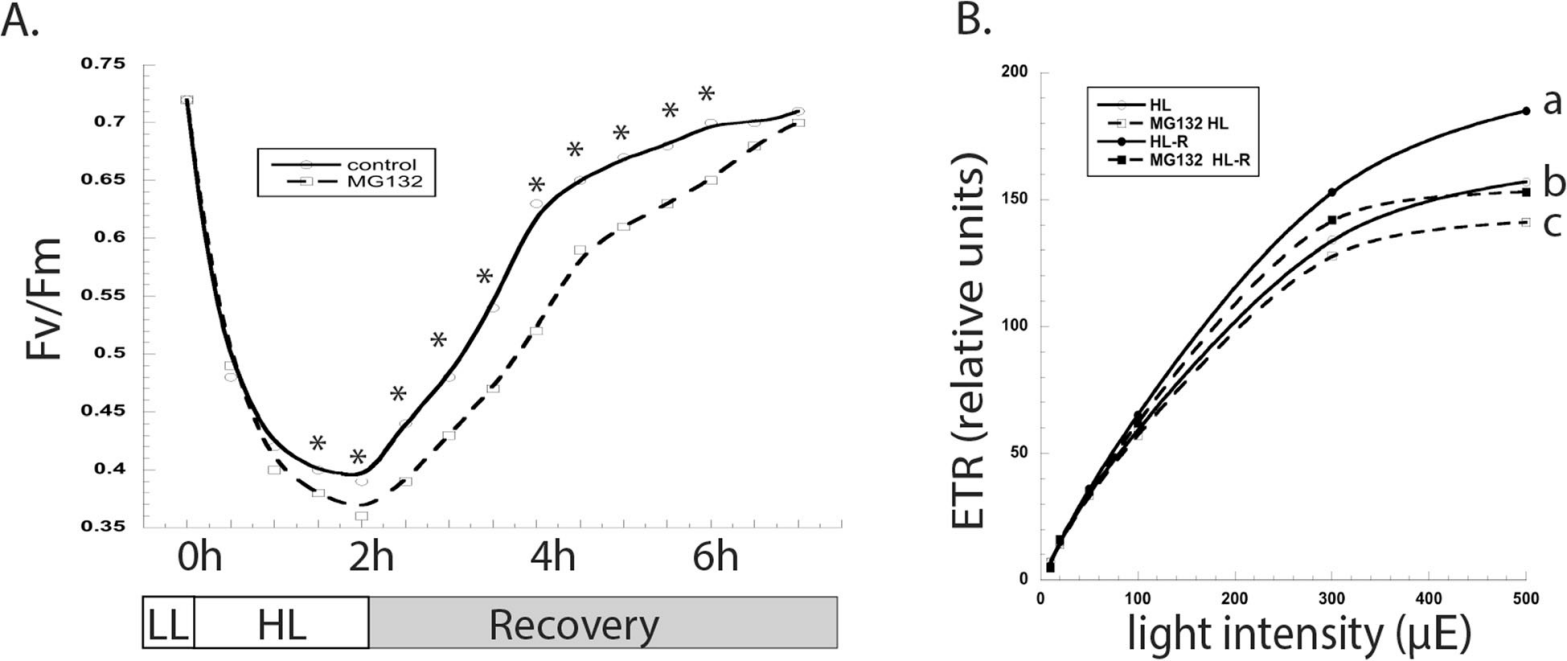
The effects of proteasome-inhibition on the ability of cells to recover from photoinhibition were evaluated using chlorophyll fluorescence. **a** Fv/Fm values were recorded prior to and during HL stress, and then during recovery; measurements were made every 30 min. **b** The ETR was estimated at the end of the 5 h recovery. Shown are the means and standard errors of five replicate cultures, which are representative of at least three other experimental replicates. Error bars are too small to plot. Asterisks represent a significant difference (*p* < 0.05) between treatments at each time point. Letters also represent a significant difference (*p* < 0.05) between treatments at each time point. LL- low light; HL- high light; R- recovery; ETR- electron transport rate

Next, we hypothesized that proteasome inhibition would alter the ability of cells to recover from photoinhibition. Cells were grown with or without MG132 for 2 h at LL, and then subjected to either an additional 2 h of LL or 2 h of HL; this was followed by recovery in the dark. Fv/Fm values recovered quicker in untreated cells compared to MG132-treated cells (Fig. 4a). After 5 h of recovery, Fv/Fm values were identical between the two cultures. Despite the recovery of Fv/Fm values, light curves revealed that proteasome inhibition attenuated the recovery of the electron transport rate at high light intensities (Fig. 4b).

**Fig. 5:**
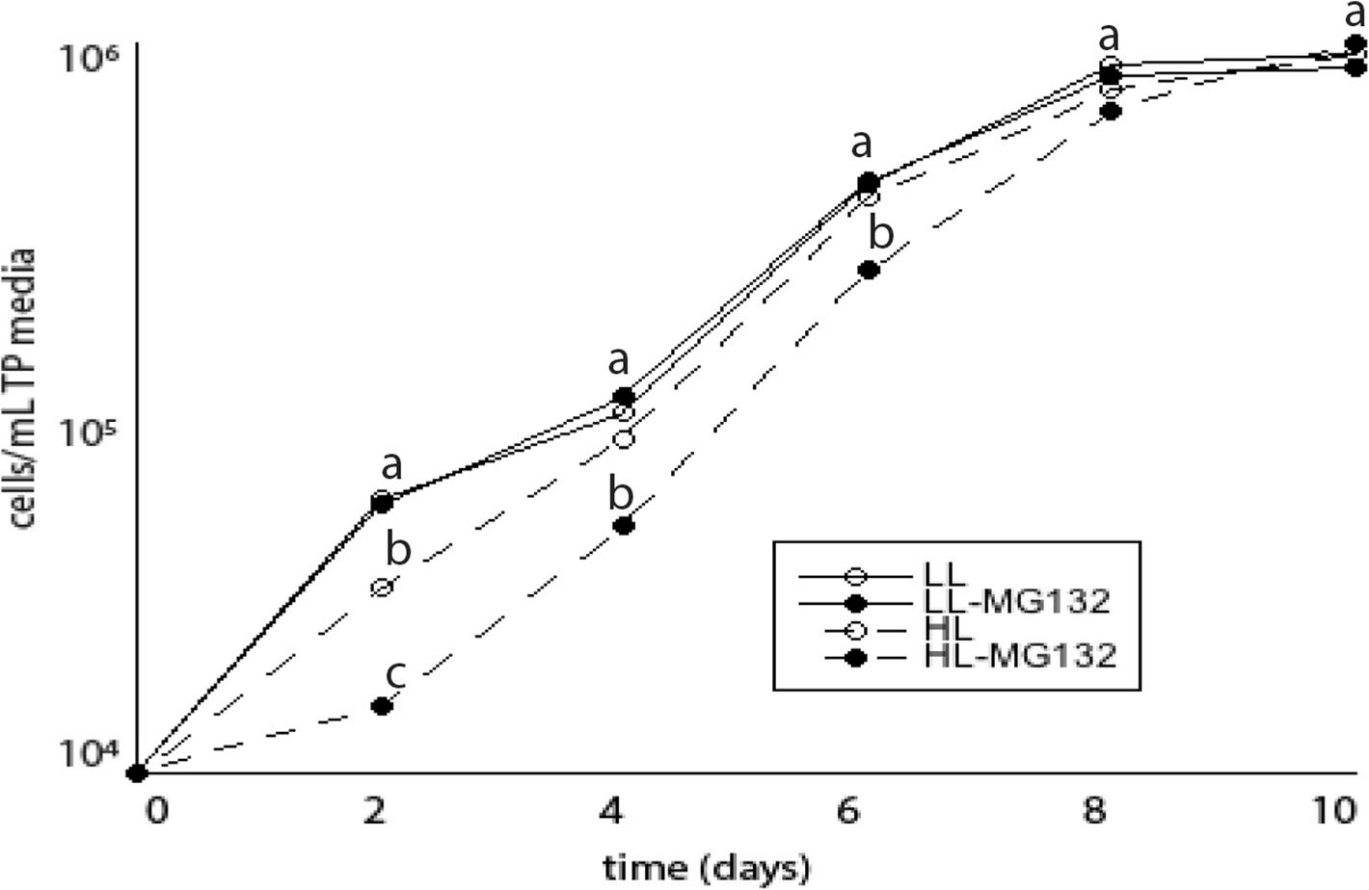
The effect of proteasome inhibition on the ability of cells to recover from photoinhibition was determined by estimating cell concentration at different time points (0–10 days). Cells treated with or without MG132 were subjected to either low light or high light for two hours, and then pelleted and transferred to TP media (10^4^ cells) and allowed to grow under LL for ten days. Shown are the means and standard errors of five replicate cultures, which are representative of at least two other experimental replicates. Error bars are too small to plot. Asterisks and different letters represent a significant difference (*p* < 0.05) between treatments at each time point. LL- low light; HL- high light; R- recovery; ETR- electron transport rate

Given that Fv/Fm values can eventually recover in photo-inhibited cells treated with MG132, we hypothesized that short-term photoinhibition in proteasome-inhibited cells has longer term consequences on the population of cells. To address this question, untreated and treated cultures were exposed to LL or HL for two hours, at which point cells were transferred into fresh TP media without MG132 and grown at LL. After 4 days, the population grew slower in proteasome-inhibited cells that had been exposed to HL compared to untreated cells (Fig. 5). However, after 8 days, there was no difference in cell concentration between the cultures.

**Fig. 6:**
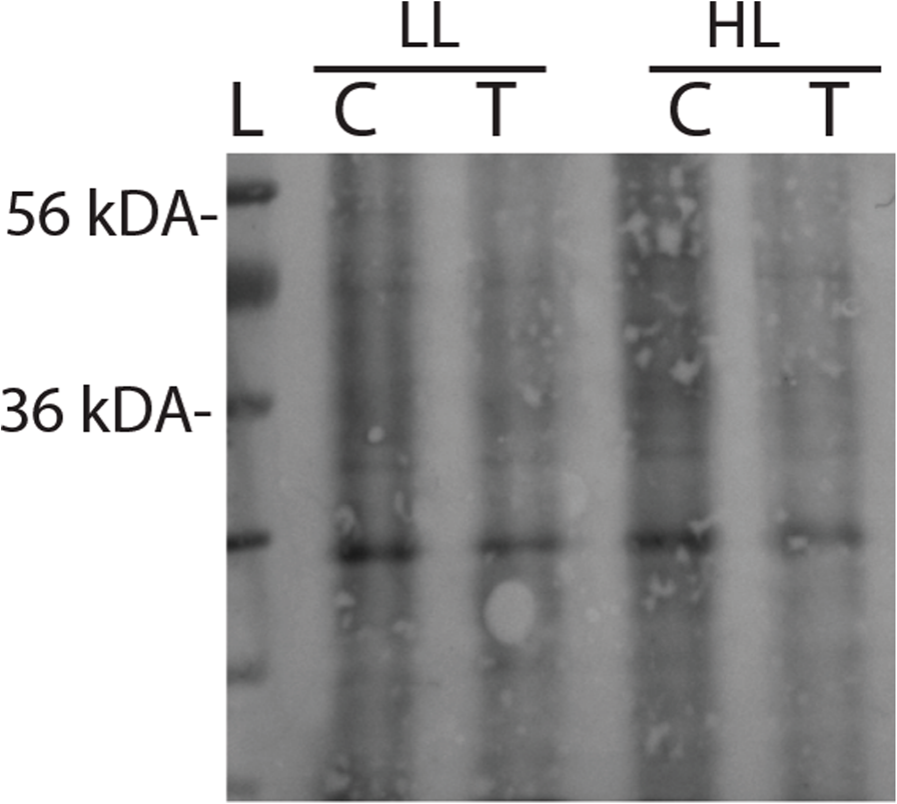
Protein synthesis was estimated in proteasome-inhibited MG132-treated cells grown under low or high light. Cells were grown without (C) or with MG132 (T) for 4 h, and then treated with puromycin for 20 min. Thirty micrograms of protein were loaded onto an SDS-PAGE gel; band intensities represented immunoreactive puromycinylated proteins, which is representative of three other blots. L- ladder; C- control; T- MG132 treatment

Our last objective was to determine why photoinhibition in proteasome-inhibited cells displayed decreased photosynthesis after 2 h of high light. Analysis of the Chlamydomonas proteasome did not reveal SP1 homologues found in Arabidopsis, *Glycine max*, *Oryza sativa*, and the moss *Physcomitrella patens* (Additional file 1: Figure S1). However, proteasome inhibition in Arabidopsis was previously shown to decrease the amino acid pool and suppress protein synthesis [25]. We hypothesized that decreased protein synthesis could contribute to decreased PSII activity during light stress, and therefore measured protein synthesis in *Chlamydamonas*. MG132 treatment decreased the synthesis of de novo proteins, as estimated from the decrease in puromycylated proteins (Fig. 6). However, protein synthesis was not affected by light treatment.

## Discussion

The recent discovery of E3 ligases in higher plants that can modulate the import of chloroplast proteins and the degradation of damaged chloroplasts has revealed a novel relationship between proteasomes and chloroplasts; these advances can likely help explain why Arabidopsis plants containing mutations in proteasome assembly have previously been reported to be sensitive to high light [23]. These discoveries have opened a new direction in chloroplast biology, and motivated us to determine if proteasome inhibition alters photosynthetic processes during photoinhibition. Our data demonstrate that proteasome inhibition caused by MG132 exacerbates photoinhibition, and dampens the ability of cells to recover from high light stress. To our knowledge, our study is the first to report a direct link between proteasome activity and protection of PSII during photoinhibition.

Compared to higher plants, using Chlamydomonas as a model has advantages to document if proteasome inhibition affects photoinhibition. Because proteasomes function in a myriad of processes, its impairment has a multitude of long-term cellular consequences that we sought to avoid by using a model that quickly absorbs MG132 in photosynthetic cells and whose viability can be tracked. Furthermore, we were able to measure both chlorophyll fluorescence and viability in the same samples soon after proteasomes were inhibited. Intriguingly, 48 h of proteasome inhibition increased size cell, which corroborates with previous reports documenting increased size of root cells in Arabidopsis treated with MG132 [26].

We focused experiments on the short-term ramifications of proteasome inhibition in cells challenged with HL. Two hours of proteasome inhibition followed by another two hours of HL impaired photosystem II, as determined by decreases in Fv/Fm and the electron transport rate. Furthermore, it took longer for proteasome-inhibited cells to recover from photoinhibition compared to untreated cells. MG132 treatment did not affect viability and chlorophyll content after four hours; therefore, using Chlamydomonas allowed us to determine that differences in photochemistry was not a result of cell death or changes in chlorophyll concentration. Additionally, in contrast to higher plants, Chlamydomonas has a single chloroplast; therefore, we can further rule out that increased photoinhibition in MG132-treated cells was a consequence of either fewer chloroplasts or an inability to remove damaged chloroplasts. Given how rapidly MG132 decreased Fv/Fm, we directly attribute the increased photoinhibition in proteasome-inhibited cells to an impairment of chloroplast processes; however, we cannot rule out pleotropic effects that might have contributed to sensitivity to HL in MG132-treated cells.

Although our experiments reveal that proteasomes protect algae against the damaging effects of photoinhibition, a precise mechanism cannot be delineated. The Chlamydomonas proteome did not reveal SP1 or PUB4 homologues; these E3 ligases in Arabdopsis [20, 22] and other higher plants respectively regulate chloroplast import or removal of damaged chloroplasts. Because proteasomes mediate transcriptional reprogramming including hormonal signaling during stress, it is possible that the increased photoinhibition observed in MG132 treated cells is a broader consequence of the inability of cells to properly respond to stress [13, 14].

It is also possible that proteasome inhibition might interfere with chloroplast proteome remodeling after photoinhibition. Proteasome inhibition decreases amino acid levels and can rapidly (2–5 h) reduce protein synthesis in Arabidopsis plants [27, 25], yeast, and animal cell lines [28]. Although photoinhibition damages many proteins in photosystem II and the cytochrome b6f complex, most of these are chloroplast-encoded proteins (e.g. D1 and PetD) that can be quickly replaced [5]. The ability to replace damaged components of PSII, such as D1, ameliorate the effects of photoinhibition. For example, Arabidopsis plants with mutations in Deg1- the protease the removes damaged D1- are susceptible to increased photoinhibition at HL [27].

Could decreased protein synthesis in MG132 restrict the ability to repair nuclear-encoded proteins in PSII? Damage to nuclear-encoded PSII proteins (e.g. Psb proteins O-S) during HL is less frequent, and therefore turnover is slower compared to plastid-encoded proteins in PSII [5]. However, damage to these proteins does occur, and reduced levels of PsbO, PsbQ, and PsbR perturb PSII efficiency [29, 30]. In tobacco, *Pseudomonas* infection decreased levels of PsbO, which was attributed to the observed increase in ROS [31]. PsbO protein is a component of the oxygen evolving complex, and levels of this protein are associated with increased protection of PSII activity during stress, such as cold treatment [32]. Intriguingly, *Pseudomonas* also releases effector molecules that can directly inhibit the proteasome [33]. Therefore, this connection exposes a possible link between proteasome inhibition and photoinhibition. Our experiments did not reveal if proteasome inhibition alters protein synthesis in plastids, or is restricted to the cytosol. However, it is possible that a drastic reduction in cytosolic protein synthesis caused by proteasome inhibition could prevent or limit how quickly nuclear proteins (e.g. PsbO, ferredoxin, *etc*) are imported into the chloroplast after stress.

In higher plants, proteasomes are known to protect against the deleterious effects caused by variety of stressors that induce ROS, including many heavy metals. Proteasome impairment was previously known to exacerbate selenium toxicity in *Chlamydomonas* [19], and this can now be extended to heavy metals and high light stress. Two hours of HL in MG132-treated cells decreased concentration of cells recovering in TP media lacking the proteasome inhibitor after 4 days; this long-term affect is likely explained by the inability of proteasome-inhibited cells to efficiently recover from photoinhibition and an unfolded protein response [34] stemming from an accumulation of misfolded proteins.

Environmental stressors can result in an accumulation of ROS, including superoxide which can directly impair proteasome activity [18]. Mounting evidence demonstrates that proteasome activity decreases during severe abiotic stress. Proteasome activity decreased almost two-fold in Chlamydomonas after 8 h of severe selenite stress; this was associated with ROS accumulation [19]. Higher plants also exhibited a decrease in proteasome activity when challenged with salt [12] and heavy metals [16]. In this context, if abiotic stress decreases proteasome activity in plants, photoinhibition could be exacerbated. Maintaining proteasome activity during stress likely enables plants to recover from photoinhibtion more quickly.

## Conclusions

This study was designed to test the hypothesis that proteasomes are involved in protecting plants against the damaging effects of photoinhibition. Using *Chlamydomonas* as a model, we demonstrate that proteasome inhibition exacerbates photoinhibition prior to observed changes in viability or changes in chlorophyll content. Therefore, we conclude that the data support the hypothesis that proteasomes have a role in ameliorating photoinhibition. Because other adverse conditions- salt, drought, and metals- can generate ROS, we additionally conclude that optimal proteasome activity in plants during stress may alleviate photoinhibition and ultimately improve crop yield.

## Methods

### Growth conditions

*Chlamydomonas reinhardtii* (wild-type strain CC-1690) was obtained from the Chlamydomonas Resource Center (University of Minnesota, MN, USA) and cultured axenically and phototrophically in tris-phosphate (TP) media lacking a carbon source under low illumination (20 μE; 16 h/8 h light dark cycle) and shaking (150 rpm) at 24 °C. pH was monitored every 2–3 days and adjusted if necessary to pH 7–7.3. Unless otherwise stated, all experiments used cells grown in TP media containing either 0.1%(v/v) dimethyl sulfoxide (DMSO) (control).

or 20 μM of MG132 dissolved in 0.1% DMSO.

### Cell viability and concentration assays

Estimates of cell viability and concentrations were performed using the Algae Count and Viability Kit according to the manufacturer’s protocol (Millipore, USA). Cells mixed with the viability probe were analyzed using a Muse® flow cytometer (Millipore, USA). Viability was determined using technical duplicates from five separate cultures, and two subsequent experimental replicates were performed to ensure consistency.

#### The effect of proteasome inhibition on cell viability

The effects of the proteasome inhibitor MG132 on cell viability and concentration were monitored at different time points, up to 48 h. Viability assays were determined in algal suspensions with an initial concentration of 10^5^ cells ml ^− 1^ of TP media containing 0, 5, 20, and 100 μM MG132. Unless otherwise stated, all subsequent experiments used a concentration of 20 μM MG132, which proved to be effective without drastically affecting viability.

#### The effect of proteasome inhibition on viability of cells exposed to metals

To demonstrate the consequences of proteasome inhibition in *Chlamydomonas* challenged with stressors that induces oxidative stress, cells were grown with or without 20 μM MG132 for 2 h and then treated with nickel, cadmium, or zinc for two days. The toxic effects of proteasome inhibition during stress was demonstrated by growing cells in 100 μM cadmium, nickel, and zinc; cultures (*n* = 5 for each treatment) were established in TP media initially containing 10^5^ cells/ml and allowed to incubate for 48 h, at which point cell viability and concentration was determined using flow cytometry.

#### The effect of proteasome inhibition on viability of cells exposed to high light stress

Prior to challenging cells with high light (HL) stress, cells were treated with or without MG132 for 2 h at LL. The effects of proteasome inhibition on the viability of cells exposed to HL stress were determined by growing *Chlamydomonas* under 1000 μE of light for 2 h and comparing to control cells grown under 20 μE, which represented low light (LL) conditions.

We also sought to monitor the long-term recovery of algal cultures treated with or without MG132 and subjected to either LL or HL; this recovery was monitored by measuring cell centration. Cells were grown for either 4 h under LL or 2 h of LL followed by 2 h of HL, at which point the culture were centrifuged (1500 *g*). Pellets were resuspended in 50 mL of TP media lackng MG132, and the cell concentration of initial cultures were diluted to 10^4^ cells ml ^− 1^. Cells were grown under LL for 10 days, and recovery was determined by measuring cell concentration every 2 days.

### Microscopy

The effect of proteasome inhibition on the volume of cells was estimated in ImageJ by analyzing images of cells captured using QCapture software attached to an Olympus BX51 microscope. Cells were treated with or without MG132 for up to 24 h, at which point cells were plated on a microscopic slide. Cell volume measurements were taken from five replicate cultures, and roughly 40–50 cells were analyzed per a replicate.

### Photosynthetic analysis

#### Chlorophyll content

Chlorophyll was measured in 2 mL of cells (10^6^ cells/ml) treated with or without MG132 grown at (*i*) 4 h at LL or (*ii*) 2 h LL followed by 2 h at HL. Cells were pelleted after 5 min of centrifugation (1500 *g*). Chlorophyll a and chlorophyll b from 3 mL of culture were extracted from pellets in 1 mL of N,N-dimethylformamide and measured spectrophotometrically [35] and subtracted for turbidity at 750 nm.

#### Chlorophyll fluorescence

Chlorophyll fluorescence was measured in cultures grown in the dark for 48 h, low light for 48 h, or high light for two h. Samples were dark-adapted for 15–20 min prior to analysis using a hand-held AquaPen chlorophyll fluorimeter (Photon System Instruments; Bratislava, Czech Republic). The ratio of variable and maximal fluorescence (Fv/Fm), which represents the maximum photochemical efficiency of photosystem II, was measured in duplicate from 5 separate cultures and calculated as described previously [36]. Electron transport rate was generated from light curves in dark adapted algae that were subjected to varying light intensities (10, 20, 50, 300, and 500 μE). After high light stress, cells recovered in the dark for 5 h. Photosynthetic recovery from photoinhibition was estimated measuring Fv/Fm every 30 min.

### Protein electrophoresis

The efficacy of proteasome inhibition was monitored by measuring the accumulation of ubiquitinated proteins as previously performed in Chlaymdomonas [19]. Cells treated with or without MG132 were grown in 100 mL of TP buffer under low light and then centrifuged at 1500 *g* at the time points indicated. Proteins were extracted in 500uL of protein extraction buffer (100 mM NaCl, 50 mMTris, pH 7.5, 0.5%(v/v) TritonX-100, and 1 mM dithiothreitol) using four repeated freeze–thaw cycles. Protein concentration was determined spectrophotometrically (A_595_) using the Bradford method. Fifty μg of denatured protein were loaded and separated on an 8% SDS-PAGE gel and transferred to a PVDF membrane. Immunoreactivity of ubiquitinated proteins in Chlaymdomonas was determined using ubiquitin antiserum (Sant Cruz Biotechnology) as previously performed [19].

Protein synthesis was estimated by using the non-radioactive SUnSET method initially developed by Schmidt et al. [37] and later modified in plants [31]. Briefly, cells with or without MG132 were grown at LL for 2 h and then transferred to either LL for 2 h or HL for 2 h and finally treated with 40 μM puromycin for 20 min; puromycin gets incorporated into nascent polypeptides and causes termination. Truncated proteins containing puromycin are detected by immunoblotting using puromycin antiserum. Thirty μg of protein were loaded onto a 15% SDS-PAGE gel and run under denaturing conditions as described above. Membranes were incubated for 2 h with the puromycin antibody (PMY-2A4) purchased from the University of Iowa, USA. The PMY-2A4 antibody was used at a 1:1000 dilution. Newly synthesized proteins containing puromycin were detected using a secondary antibody conjugated to alkaline phosphatase (1:10,000 dilution for 45 min).

### Statistical and bioinformatic analysis

Statistical analyses were performed using the Kaleida-Graph software program (Synergy Software), and included Student t-tests and analysis of variance (ANOVA).

Searches for homologues of the SPI E3 ligase in Chlamydomonas were performed in using BlastP (https://www.ncbi.nlm.nih.gov/). Alignment of the SPI in Arabidopsis, *Glycine max*, *Oryza sativa*, and the moss *Physcomitrella patens* were performed in Clustal Omega.

## Supplementary information

**Additional file 1: Figure S1.** Chlamydomonas does not possess the SP1 E3 ligase found in land plants. Alignment (top) and evolutionary divergence (bottom) of SP1 in Chlamydomonas, Physcomitrella patens, Oryza sativa, Arabidopsis, and Glycine max. #- presence of SP1; −absence of SP1.

## Data Availability

The datasets used and/or analyzed during the current study are available from the corresponding author on reasonable request.

## Abbreviations

ETR: Electron Transport rate
HL: High light
LL: Low light
PSII: Photosystem II
ROS: Reactive oxygen species
TOC: Translocon at the outer envelope of the chloroplast
UPS: Ubiquitin proteasome pathway

## References

[1] Apel K, Hirt H (2004). Reactive oxygen species: metabolism, oxidative stress, and signal transduction. Annu Rev Plant Biol.

[2] Pospíšil P (2016). Production of reactive oxygen species by photosystem II as a response to light and temperature stress. Front Plant Sci.

[3] Balk J, Pilon M (2011). Ancient and essential: the assembly of iron-sulfur clusters in plants. Trends Plant Sci.

[4] Fisher B, Yarmolinsky D, Abdel-Ghany S, Pilon M, Pilon-Smits EA, Sagi M, Van Hoewyk D (2016). Superoxide generated from the glutathione-mediated reduction of selenite damages the iron-sulfur cluster of chloroplastic ferredoxin. Plant Physiol Biochem.

[5] Li L, Aro EM, Millar AH (2018). Mechanisms of photodamage and protein turnover in photoinhibition. Trends Plant Sci.

[6] Erickson E, Wakao S, Niyogi KK (2015). Light stress and photoprotection in *Chlamydomonas reinhardtii*. Plant J.

[7] Richardson LG, Singhal R, Schnell DJ (2017). The integration of chloroplast protein targeting with plant developmental and stress responses. BMC Biol.

[8] Liu JX, Howell SH (2010). Endoplasmic reticulum protein quality control and its relationship to environmental stress responses in plants. Plant Cell.

[9] Smalle J, Vierstra RD (2004). The ubiquitin 26S proteasome proteolytic pathway. Annu Rev Plant Biol.

[10] Genschik P, Criqui MC, Parmentier Y, Derevier A, Fleck J (1998). Cell cycle–dependent proteolysis in plants: identification of the destruction box pathway and metaphase arrest produced by the proteasome inhibitor MG132. Plant Cell.

[11] Yates G, Sadanandom A (2013). Ubiquitination in plant nutrient utilization. Front Plant Sci.

[12] Wang S, Kurepa J, Hashimoto T, Smalle JA (2011). Salt stress–induced disassembly of Arabidopsis cortical microtubule arrays involves 26S proteasome-dependent degradation of SPIRAL1. Plant Cell.

[13] Santner A, Estelle M (2009). Recent advances and emerging trends in plant hormone signaling. Nature.

[14] Lyzenga WJ, Stone SL (2012). Abiotic stress tolerance mediated by protein ubiquitination. J Exp Bot.

[15] Sabbagh M, Van Hoewyk D (2012). Malformed selenoproteins are removed by the ubiquitin-proteasome pathway in *Stanleya pinnata*. Plant Cell Physiol.

[16] Pena LB, Zawoznik MS, Tomaro ML, Gallego SM (2008). Heavy metals effects on proteolytic system in sunflower leaves. Chemosphere.

[17] Yabuta Y, Osada R, Morishita T, Nishizawa-Yokoi A, Tamoi M, Maruta T, Shigeoka S (2011). Involvement of Arabidopsis NAC transcription factor in the regulation of 20S and 26S proteasomes. Plant Sci.

[18] Wang X, Yen J, Kaiser P, Huang L (2010). Regulation of the 26S proteasome complex during oxidative stress. Sci Signal.

[19] Vallentine P., Hung C.-Y., Xie J., Van Hoewyk D. (2014). The ubiquitin-proteasome pathway protects Chlamydomonas reinhardtii against selenite toxicity, but is impaired as reactive oxygen species accumulate. AoB PLANTS.

[20] Ling Q, Huang W, Baldwin A, Jarvis P (2012). Chloroplast biogenesis is regulated by direct action of the ubiquitin-proteasome system. Science.

[21] Ling Q, Jarvis P (2015). Regulation of chloroplast protein import by the ubiquitin E3 ligase SP1 is important for stress tolerance in plants. Curr Biol.

[22] Woodson JD, Joens MS, Sinson AB, Gilkerson J, Salomé PA, Weigel D, Fitzpatrick JA, Chory J (2015). Ubiquitin facilitates a quality-control pathway that removes damged chloroplasts. Science.

[23] Lee KH, Marshall RS, Slivicke LM, Vierstra RD (2012). Genetic analyses of the Arabidopsis 26S proteasome regulatory particle reveal its importance during light stress and a specific role for the N-terminus of RPT2 in development. Plant Signal Behav.

[24] Fischer BB, Krieger-Liszkay A, Eggen RI (2005). Oxidative stress induced by the photosensitizers neutral red (type I) or rose bengal (type II) in the light causes different molecular responses in *Chlamydomonas* reinhardtii. Plant Sci.

[25] Van Hoewyk D. Use of the non-radioactive SUnSET method to detect decreased protein synthesis in proteasome inhibited Arabidopsis roots. Plant Methods. 2016;12:20. 10.1186/s13007-016-0120-z PMC4794914 26989430

[26] Sheng Xianyong, Wei Qian, Jiang Liping, Li Xue, Gao Yuan, Wang Li (2012). Different Degree in Proteasome Malfunction Has Various Effects on Root Growth Possibly through Preventing Cell Division and Promoting Autophagic Vacuolization. PLoS ONE.

[27] Kapri-Pardes Einat, Naveh Leah, Adam Zach (2007). The Thylakoid Lumen Protease Deg1 Is Involved in the Repair of Photosystem II from Photoinhibition in Arabidopsis. The Plant Cell.

[28] Ding Q, Dimayuga E, Markesbery WR, Keller JN (2006). Proteasome inhibition induces reversible impairments in protein synthesis. FASEB J.

[29] Allahverdiyeva Y, Suorsa M, Rossi F, Pavesi A, Kater MM, Antonacci A, Tadini L, Pribil M, Schneider A, Wanner G, Leister D (2013). Arabidopsis plants lacking PsbQ and PsbR subunits of the oxygen-evolving complex show altered PSII super-complex organization and short-term adaptive mechanisms. Plant J.

[30] Henmi T, Miyao M, Yamamoto Y (2004). Release and reactive-oxygen-mediated damage of the oxygen-evolving complex subunits of PSII during photoinhibition. Plant Cell Physiol.

[31] Cheng DD, Zhang ZS, Sun XB, Zhao M, Sun GY, Chow WS (2016). Photoinhibition and photoinhibition-like damage to the photosynthetic apparatus in tobacco leaves induced by pseudomonas syringae pv. Tabaci under light and dark conditions. BMC Plant Biol.

[32] Pawłowicz I, Kosmala A, Rapacz M (2012). Expression pattern of the psbO gene and its involvement in acclimation of the photosynthetic apparatus during abiotic stresses in *Festuca arundinacea* and *F. pratensis*. Acta Physiol Plant.

[33] Groll M, Schellenberg B, Bachmann AS, Archer CR, Huber R, Powell TK (2008). A plant pathogen virulence factor inhibits the eukaryotic proteasome by a novel mechanism. Nature.

[34] Bush KT, Goldberg AL, Nigam SK (1997). Proteasome inhibition leads to a heat-shock response, induction of endoplasmic reticulum chaperones, and thermotolerance. J Biol Chem.

[35] Porra RJ, Thompson WA, Kriedemann PE (1989). Determination of accurate extinction coefficients and simultaneous equations for assaying chlorophylls a and b extracted with four different solvents: verification of the concentration of chlorophyll standards by atomic absorption spectroscopy. Biochim Biophys Acta.

[36] Maxwell K, Johnson GN (2000). Chlorophyll fluorescence—a practical guide. J Exp Bot.

[37] Schmidt EK, Clavarino G, Ceppi M, Pierre P (2009). SUnSET, a nonradioactive method to monitor protein synthesis. Nat Methods.

