# Supplementary material for: Proteasome inhibition rapidly exacerbates photoinhibition and impedes recovery during high light stress in Chlamydomonas reinhardtii
Source: BMC Plant Biol. 2020 Jan 13;20:22. doi: 10.1186/s12870-020-2236-6 (PMC6958727; doi:10.1186/s12870-020-2236-6)

SI Figure 1. Chlamydomonas does not possess the SP1 E3 ligase found in land plants. Alignment (top) and evolutionary divergence (bottom) of SP1 in Chlamydomonas, Physcomitrella patens, Oryza sativa, Arabidopsis, and Glycine max. #- presence of SP1; □-absence of SP1.

```
Physcomitrella      -MLSWGGITLCLSGAALYCLSRNTGRDALNLRSIERVNQLKDLAILLESACKVVPLVVTV        59
Oryza               MLIPWGGVGCCLSAAALYLLGRSSGRDAEVLRSVARAGSTKDLAAILDTASKVLPLVVAV        60
Arabidopsis         -MIPWGGVTCCLSAAALYLLGRSSGRDAEVLETVTRVNQLKELAQLLELDSKILPFIVAV        59
Glycine             -MIPWGGLSCCLSAAALYLLGRSSGRDAEILKSVTRVNQLKELAQLL--DAEILPLIVTI        57
                     :: ***:  ***.**** *.*.:****  *.:: *... *:** :*   .:::*::*::

Physcomitrella      AGRVGSETPIACEHSSLRGVILEETAEQHFLKHNDTGSWIQDSALMLSISKEVPWYLEDG        119
Oryza               SGRVGSDTPLICQQSGMRGVIVEETAEQHFLKHNDAGSWIQDSAVMLSVSKEVPWYLDDG        120
Arabidopsis         SGRVGSETPIKCEHSGIRGVIVEETAEQHFLKHNETGSWVQDSALMLSMSKEVPWFLDDG        119
Glycine             SGRVSSETPINCEFSGLRGVIVEETAEQHFLKHNDAGSWIQDSALMLSMSKEVPWYLDDG        117
                    :***.*:**: *: *.:****:************::***:****:***:******:*:**

Physcomitrella      TGRVYIVGARNAAGMELTVASEVFEESGRSLVRGTLDYLQGLKMLGVKRVERVLPTGTNL        179
Oryza               TGRVFVVGARGAAGLVLTVASEVFEESGRTLVRGTLDYLQGLKMLGVKRTERVLPTGTSL        180
Arabidopsis         TSRVHVMGARGATGFALTVGSEVFEESGRSLVRGTLDYLQGLKMLGVKRIERVLPTGIPL        179
Glycine             TDRVHVVGARGAAGFALPVGSEAFEESGRSLVRGTLDYLQGLKMLGVKRIERVLPVGTSL        177
                    *.**.::***.*:*: * *.**.******:****************** *****.*  *

Physcomitrella      TVVGEAVQDDRGLIRIQKPNKGPFYVTPKSLDQLVANLGRWSRWYKYMSLGFTIVGIYFI        239
Oryza               TVVGEAIKDDVGTIRIQRPHKGPFYVSPKSIDQLIMNLGKWAKLYQLASMGFAAFGVFLL        240
Arabidopsis         TIVGEAVKDDIGEFRIQKPDRGPFYVSSKSLDQLISNLGKWSRLYKYASMGFTVLGVFLI        239
Glycine             TVVGEAAKDDVGAFRIQRPHKGPFYVSPKTIDQLIANLGKWARWYKYASMGLTVFGAYLI        237
                    *:**** :** * :***:*.:*****: *::***: ***:*:: *:  *:*:: .* :::

Physcomitrella      TSHAIKHFMERRRREALHRRVMEAAALRQASQREGGDGDMGDVTSHPLDDSVDTSQKKDR        299
Oryza               AKRALQHFLERKRRHELQKRCVLTNHLF----------------------GFRRTCYSHL        278
Arabidopsis         TKHVIDSVLERRRRRQLQKRVLDAAAKRAELESEGIH-----------W-----------        277
Glycine             AKHAIRYILERRRRSELQRRVLAAAAKKSGQNNDVEK-----------ADGLSDGVKKDR        286
                    :.:.:  .:**:**  *::* : :

Physcomitrella      GTPDLCVICLEQDYNAVFLPCGHMCCCTSCSAQLTSCPLCRRHIDKFVKTYRH       352
Oryza               HSSVVCSLC--------------------------------------------       287
Arabidopsis         -----------------------------------------------------       277
Glycine             LMPDLCVICLEQEYNAVFVPCGHMCCCTTCSSHLTNCPLCRRQIEKVVKTFRH       339
```

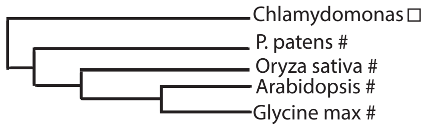

Supplement: Supplementary file 1 — Additional file 1: Figure S1. Chlamydomonas does not possess the SP1 E3 ligase found in land plants. Alignment (top) and evolutionary divergence (bottom) of SP1 in Chlamydomonas, Physcomitrella patens, Oryza sativa, Arabidopsis, and Glycine max. #- presence of SP1; −absence of SP1. [file 12870_2020_2236_MOESM1_ESM.pdf]
